# Super-resolution microscopy unveils transmembrane domain-mediated internalization of cross-reacting material 197 into diphtheria toxin-resistant mouse J774A.1 cells and primary rat fibroblasts in vitro

**DOI:** 10.1007/s00204-020-02731-4

**Published:** 2020-04-08

**Authors:** Maximilian Fellermann, Fanny Wondany, Stefan Carle, Julia Nemeth, Tanmay Sadhanasatish, Manfred Frick, Holger Barth, Jens Michaelis

**Affiliations:** 1 Institute of Pharmacology and Toxicology, Ulm University Medical Center, Albert-Einstein-Allee 11, 89081 Ulm, Germany; 2 Institute of Biophysics, Ulm University, Albert-Einstein-Allee 11, 89081 Ulm, Germany; 3 Institute of General Physiology, Ulm University, Albert-Einstein-Allee 11, 89081 Ulm, Germany

**Keywords:** Cross-reacting material 197 (CRM197), Diphtheria toxin (DT), Resistance, Murine, Heparin-binding epidermal growth factor-like growth factor precursor (HB EGF), STED super-resolution optical microscopy

## Abstract

Diphtheria toxin (DT) efficiently inhibits protein synthesis in human cells, resulting in severe disease diphtheria. The sensitivity towards DT varies between mammalian species. Mice and rats are resistant to DT. However, the reason underlying this insensitivity is controversially discussed and not well understood. Therefore, we investigated the steps of DT uptake, i.e. receptor binding and internalization into mouse J774A.1 macrophages and primary rat fibroblasts. We exploited the non-toxic DT-mutant cross-reacting material 197 (CRM197) and three additional receptor binding-deficient mutants (250 nM each) to investigate binding to cell surface and internalization into murine cells via flow cytometry and stimulated emission depletion (STED) super-resolution optical microscopy. Dual-color STED imaging unveiled CRM197 interacting with the murine precursor of the heparin-binding epidermal growth factor-like growth factor (HB-EGF). Moreover, we identified CRM197’s transmembrane domain as an additional HB-EGF binding site, which is also involved in the receptor-mediated internalization into murine cells. However, we do not find evidence for translocation of the catalytically active subunit (DTA) into the cytosol when 250 nM DT were applied. In conclusion, we provide evidence that the resistance of murine cells to DT is caused by an insufficiency of DTA to escape from endosomes and reach the cytosol. Possibly, a higher affinity interaction of DT and the HB-EGF is required for translocation, which highlights the role of the receptor in the endosomes during the translocation step. We extend the current knowledge about cellular uptake of the medically relevant DT and CRM197.

**Electronic supplementary material:**

The online version of this article (10.1007/s00204-020-02731-4) contains supplementary material, which is available to authorized users.

## Introduction

Diphtheria toxin (DT) is a single protein (58 kDa) produced from *Corynebacterium diphtheria* (Pappenheimer 1977; Greenfield et al. 1983). In the crystal structure of DT, three distinct domains can be distinguished (Choe et al. 1992; Bennett and Eisenberg 1994). The catalytic (C-) domain is located in the N-terminal part of DT. The transmembrane (T-) domain in the middle of DT connects the C-domain with the receptor-binding (R-) domain at the C-terminus (Choe et al. 1992; Bennett and Eisenberg 1994). The R-domain binds a specific cell surface receptor identified as membrane-anchored precursor of the heparin-binding epidermal growth factor-like growth factor (HB-EGF) (Naglich et al. 1992; Mitamura et al. 1995). The affinity of this interaction is enhanced by the presence of the CD9 antigen (DRAP27/CD9), which acts as co-receptor for DT (Iwamoto et al. 1991, 1994). However, the presence of CD9 alone is not enough to mediate DT-sensitivity (Mitamura et al. 1992). DT is internalized into cells via receptor-mediated endocytosis (Keen et al. 1982). During this process, furin proteases cleave DT between the C- and T-domain into two fragments named DTA and DTB (Tsuneoka et al. 1993). DTA contains the catalytic domain, while DTB consists of the T- and R-domain (Choe et al. 1992). After internalization and cleavage, the early endosomes start to mature and their lumen becomes acidified. Thereby, DTB undergoes a structural rearrangement, probably leading to the insertion of the T-domain into the endosomal membrane, what contributes to the translocation of DTA into the cytosol (Choe et al. 1992; Silverman et al. 1994). Subsequently, DTA catalyzes the ADP-ribosylation of the eukaryotic elongation factor 2 (eEF2). In this reaction, ADP-ribose from NAD^+^ is covalently transferred to eEF2, which is thereby inactivated resulting in the inhibition of protein synthesis and cell death (Goor et al. 1967; Honjo et al. 1968; Pappenheimer 1977; Kaneda et al. 1984). There are several DT mutants known, in which one of these steps (binding, uptake or ADP-ribosylation) is specifically inhibited. Cross-reacting material 197 (CRM197) carries a mutation (G52E) in the C-domain blocking the ADP-ribosylation reaction and is, therefore, non-toxic (Uchida et al. 1972, 1973; Giannini et al. 1984; Malito et al. 2012). Nevertheless, its crystal structure is nearly identical to that of DT (Malito et al. 2012). Therefore, it is an ideal candidate to investigate DT binding and internalization without harming the cells.

DT efficiently intoxicates the cells of many mammalian species including humans, rabbits, and guinea pigs as well as several birds (Pappenheimer 1977). In contrast, murine cells, i.e. mice and rats remain insensitive to DT (Singer 1945; Moehring and Moehring 1968; Collier 1975; Chang and Neville 1978). However, the reason for the murine DT-resistance is controversially discussed (Manoilov et al. 2018). Notably, the DT-receptor HB-EGF is expressed by DT-sensitive mammalian cells as well as by DT-resistant murine cells (Abraham et al. 1993). Nevertheless, the HB-EGF from primates differs from that of rodents in its primary structure (Abraham et al. 1993). Some data suggest that due to these differences in the amino acid sequences, DT is unable to bind to the murine HB-EGF (Collier 1975; Boquet and Pappenheimer 1976; Proia et al. 1979; Naglich et al. 1992; Mitamura et al. 1995) or binds only with a reduced affinity (Heagy and Neville 1981). Hence, resistance may be caused due to a failure of DT-binding and/or DT-internalization. In contrast, other groups showed that the binding of DT to murine cells is not, or not impaired sufficiently to explain the DT-resistance (Chang and Neville 1978; Heagy and Neville 1981; Didsbury et al. 1983; Manoilov et al. 2018). Furthermore, the successful internalization of DT into endosomes of murine cells (Keen et al. 1982; El Hage et al. 2008; Manoilov et al. 2018) and furin cleavage of DT in rat cell endosomes (El Hage et al. 2008) was reported. Based on this data, the reason of murine DT-resistance could be caused by steps after receptor binding and internalization of DT (Keen et al. 1982; Didsbury et al. 1983; El Hage et al. 2008; Manoilov et al. 2018). In vitro, the ADP-ribosylation of murine eEF2 by DTA is comparable to DT-sensitive species (Goor et al. 1967; Moehring and Moehring 1968; Park et al. 2018). Hence, one hypothesis is that insufficient transport of DTA into the cytosol of rodents is the reason for the DT-resistance (Goor et al. 1967; Moehring and Moehring 1968).

Despite the controversy and murine DT-resistance, mouse-models are frequently used to investigate the function of CRM197 as vaccine carrier (Stickings et al. 2008; Fiorino et al. 2012; Mishra et al. 2018) or as a coating on nanoparticles to mediate blood–brain barrier crossing (Tosi et al. 2015). Therefore, we re-investigated binding to cell surface and internalization into DT-resistant murine cells using novel technologies, namely super-resolution optical microscopy giving us qualitative and quantitative insight into receptor binding and cellular uptake of different non-toxic DT mutants (e.g. CRM197). We observed co-localization with the murine HB-EGF receptor during cell surface-binding and with early endosomal antigen 1 (EEA1) after internalization, proving that the uptake uses receptor-mediated endocytosis. Comparable results were obtained for mutants known to be deficient in receptor binding or mutants lacking the complete R-domain. However, this interaction can be blocked by deleting the T-domain. This indicates that the observed interaction takes place between DT’s T-domain and the murine HB-EGF. Possibly, the T-domain supports the B-domain in binding to the HB-EGF or this interaction is indirect and involves a co-receptor.

In conclusion, our results implicate that murine HB-EGF is involved (directly or indirectly) in the internalization of DT into mouse or rat early endosomes. However, DTA does not efficiently translocate from endosomes into the cytosol of murine cells, which is probably the reason for the DT-resistance.

## Materials and methods

The manufacturers of the used materials are listed in the supplements as well as more detailed method descriptions.

### Cell culture

Murine macrophage-like J774A.1 cells were cultivated in DMEM (10% FCS, 1% penicillin–streptomycin, 1 nM sodium pyruvate). Human HeLa cervix carcinoma cells were cultivated in Minimum Essential Medium Eagle (MEM with 10% FCS, 1% penicillin–streptomycin, 1 mM sodium pyruvate, 2 mM l-glutamine and 0.1 mM non-essential amino acids). Cells were maintained at 37 °C and 5% CO_2_. Subconfluent cells were passaged. The J774A.1 and HeLa cells were treated 1 or 2 days after seeding.

### Isolation of primary rat lung fibroblasts

Primary lung fibroblasts were isolated from 12 to 14-week-old male Sprague–Dawley rats according to a modified protocol described by Dobbs et al. (1986). In short, rats were anesthetized with ketamin (10%) and xylazil (2%), and injected with heparin (400 IU/kg). Lungs were perfused, removed, washed and dissected. The tissue was incubated with 0.5 mg/mL elastase and 0.05 mg/mL at 37 °C for 30 min. Then, 2 mg/mL DNAse was added and the tissue was minced with sharp scissors into bits of about 1 mm^3^. The enzymatic reaction was stopped by adding FCS (37 °C, 2 min). The digested tissue was filtered through gauze and nylon meshes (mesh sizes: 100, 70 and 40 μm) and the cell filtrate was centrifuged for 8 min at 130 rcf. For purification of primary fibroblasts, cell suspensions were depleted of leucocytes using anti-CD45 MicroBeads before fibroblasts were isolated using anti- CD90.1 MicroBeads according to manufacturer’s instructions.

Cells were seeded and maintained in MucilAir supplemented with 25.6 μg/mL Gentamicin at 37 °C, 5% CO2, and 95% humidity. All animal experiments were performed according to approved guidelines. The primary rat fibroblasts were treated with the DT-variants on day six or seven after seeding when they show characteristic elongated fibroblast morphology.

### Molecular cloning of ^His_^eGFP-labeled proteins

**Fig. 1:**
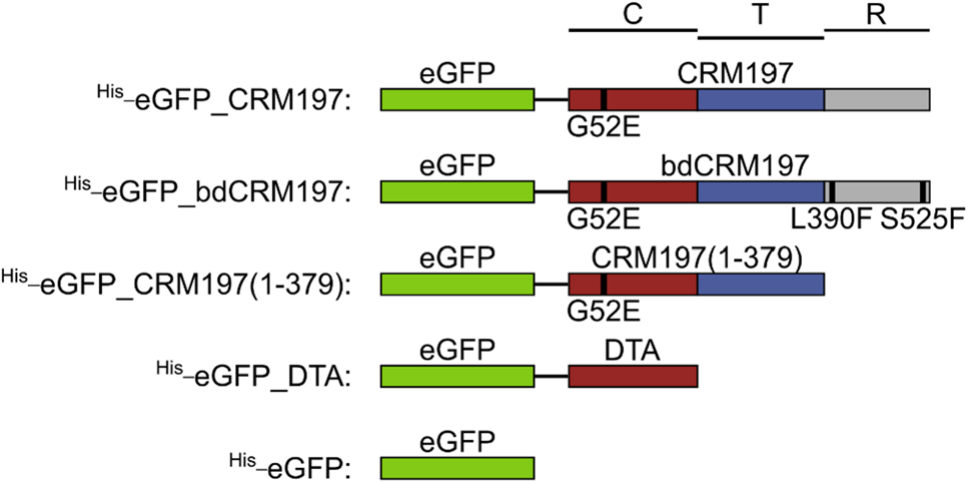
Cartoon of the cloned eGFP-constructs. The different proteins are linked with a GSG-linker. The different CRM197 domains are highlighted (C-domain: red, T-domain: blue, R-domain: gray). Point mutations are marked below and within the constructs (amino acid exchange and black vertical bar) (Color figure online)

The basic plasmids coding for CRM197 and eGFP were modified using traditional cloning techniques, the In-Fusion^®^ cloning kit or side-directed mutagenesis (see Fig. 1 and method description in the supplements).

### Recombinant expression and purification of His-tagged proteins

The His-tagged proteins were expressed in *Escherichia coli* Shuffle^®^ T7 Express in LB-medium containing 0.5 mM IPTG. After harvesting and lysis of the cells the His-tagged proteins were purified using the ÄKTA^®^ FPLC system in combination with 1 mL Ni–NTA columns (see supplements). SDS-PAGE and Western blot detection was performed to confirm the identity and purity of the purified proteins (see Fig. S 3).

### Binding and internalization analysis in flow cytometry

J774A.1 cells were treated with PBS or 500 µg/mL pronase in PBS for 30 min at 37 °C. Afterwards, the J774A.1 cells were mechanically detached with a cell scraper. HeLa cells were either incubated with 25 mM EDTA in PBS or 500 µg/mL pronase in PBS for 30 min at 37 °C. To fully remove the pronase or EDTA, the detached cells were suspended in 10% FCS containing MEM-medium and washed twice with PBS. The cells were separated by pipetting them up and down. $2 \cdot 10^{5}$ cells were incubated with the respective eGFP-labeled proteins at the indicated temperature in FCS-free medium. Subsequently, the cells were centrifuged (500 rcf at 4 °C for 3 min) and washed twice with ice-cooled PBS. The cell-associated fluorescence at an excitation wavelength of 488 nm was measured in a flow cytometer. 10^4^ cells were analyzed for each condition. For the cellular uptake investigations, extracellular eGFP-signals were quenched with 50 ng/µL Trypan Blue. The median fluorescence intensities were calculated using the flowing software 2.5.1. The means of one triplicate (*n* = 3) ± standard deviations (SD) were normalized to the negative control.

### Sample preparation and immunostaining for microscopy experiments

Cells were seeded in 8-Well ibidi µ-Slide and incubated with respective proteins (250 nM) at the indicated temperature and time in FCS-free medium. Cells were fixated with paraformaldehyde, permeabilized with TritonX-100 and blocked with BSA. Primary antibodies were incubated overnight at 4 °C. Subsequent second antibody incubation was performed for 1 h at RT. Prior to imaging, PBS was exchanged for 2,2′-thiodiethanol.

### Confocal and STED super-resolution microscopy

Images were captured with a home-built dual-color 3D-STED microscope (Osseforth et al. 2014). Typically, an average power of ~ 0.8 µW for each excitation beam and ~ 1.3 mW for each depletion beam was used. Confocal images were captured at a pixel size of 50 nm and a dwell time of 200 µsec with a typical peak photon number of ~ 200 counts. STED images were captured at a pixel size of 12.5 nm and a dwell time of 300 µsec with a typical peak photon number of ~ 150 counts. Images were analyzed by ImageJ 1.52n; For better visualization, a Gaussian blur of *σ* = 1, and a channel intensity threshold > 40 counts on confocal images and > 20 counts on STED images was applied in each channel.

### Reproducibility and statistics

All experiments were performed three times under comparable conditions. The most representative results are shown. The results are depicted as mean ± standard deviation (± SD). Green spots in STED images were automatically quantified with a programed search algorithm in Python 3.7.

## Results and discussion

To investigate the receptor-mediated internalization of DT into murine cells the advantages of different fluorescence-based methods were combined. While flow cytometry offers the possibility to quantitatively compare different mutants and cell types in a high-throughput manner, STED super-resolution optical microscopy enables us to capture the details in the binding and uptake process thus giving direct insight into mechanistic principles. Instead of wild-type DT, we used the well-established non-toxic DT-mutant CRM197 (Uchida et al. 1972, 1973) in our experiments. The usage of CRM197, which has only a single point mutation (G52E) in the catalytic domain (DTA) (Giannini et al. 1984; Malito et al. 2012) has several advantages: (1) in contrast to DT, CRM197 does not induce cytotoxic effects and changes in cell-morphology (e.g. cell-rounding), which is essential to analyze protein uptake and intracellular trafficking by super-resolution microscopy; (2) cloning and expression of wild-type DT is classified as hazardous and not allowed without special safety conditions and, therefore, no recombinant DT could be produced. Additionally, the crystal structure of CRM197 is nearly identical to that of DT, except for a flexible loop in the NAD^+^ binding pocket (Malito et al. 2012). Therefore, CRM197 is the ideal mutant to investigate binding and the cellular uptake of DT. Fusion of a green fluorescent protein (eGFP) to the N-terminus of CRM197 (^His_^eGFP_CRM197) enabled us to quantify the binding of CRM197 to cells and to follow the internalization of CRM197 (Fig. 1). Based on this fusion protein, two mutants were generated (Fig. 1). One of them carries two point mutations in the receptor-binding domain (L390F, S525F), which were previously found in CRM107 (Greenfield et al. 1987). It has been reported that due to the amino acid exchanges the binding affinity for the HB-EGF is reduced 8000-fold compared to DT (Greenfield et al. 1987). In the following, this mutant is called binding-deficient CRM197 (^His_^eGFP_bdCRM197). In the second mutant, the binding domain was deleted via insertion of two stop codons. Thereby, only the amino acids 1-379 of CRM197 were expressed (^His_^eGFP_CRM197(1-379)). Due to these alterations in the receptor-binding domain, the mutants are expected to be unable to bind to HB-EGF (^His_^eGFP_CRM197(1-379)) or to bind with much lower affinity (^His_^eGFP_bdCRM197) (Greenfield et al. 1987). SDS-PAGE and Western blot detection was performed to confirm the identity and purity of the purified proteins (Fig. S 3).

### Quantitative analysis of binding and internalization of CRM197 using flow cytometry

**Fig. 2:**
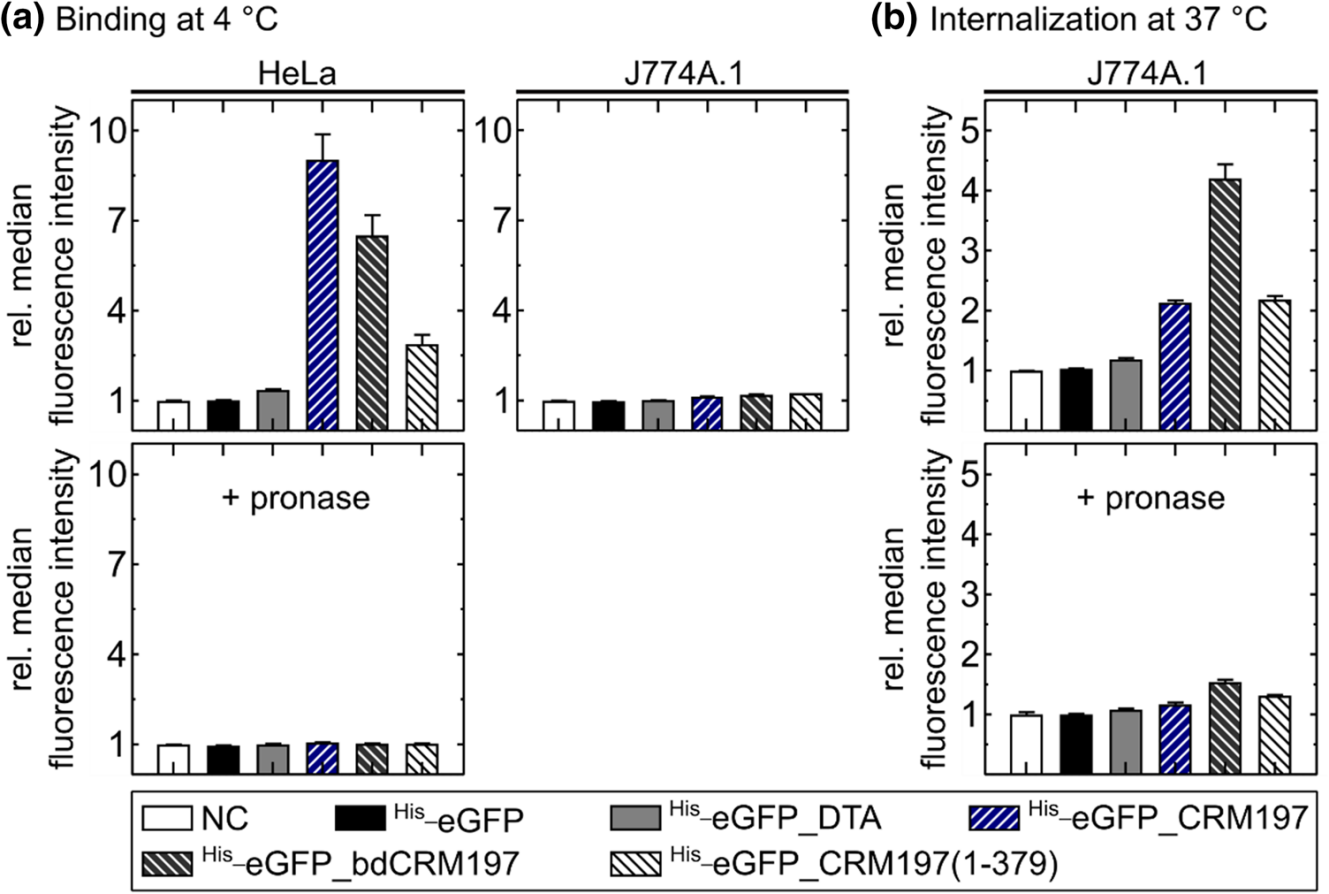
Flow cytometric analysis of cellular binding and internalization of CRM197 and mutants. The diagrams show the relative median fluorescence intensity at 488 nm excitation (gated for the main population and normalized to the PBS-treated negative control (NC)). Therefore, detached cells were treated with PBS (NC), ^His_^eGFP, ^His_^eGFP_DTA ^His_^eGFP_CRM197, ^His_^eGFP_bdCRM197, ^His_^eGFP_CRM197(1-379) (250 nM each) for 30 min. **a** Human HeLa (left panels) and murine J774A.1 (right panel) were treated at 4 °C. As a control for specific binding to cell surface proteins, HeLa cells were pre-incubated with 500 µg/mL pronase for 30 min at 37 °C (left lower panel). **b** Murine J774A.1 cells were incubated with ^His_^eGFP proteins at 37 °C for 30 min. To quench extracellular eGFP signals, 50 µg/mL trypan blue (TB) was added directly before the analysis in the flow cytometer. As a control for specific internalization via interaction with a proteinogenic cell surface receptor, J774A.1 cells were pre-incubated with 500 µg/mL pronase at 37 °C for 30 min to degrade all surface proteins (lower panel). **a**, **b** The values are given as mean of three measurements ± standard deviation (SD) obtained in one representative experiment

The binding of all generated CRM197 proteins to DT-sensitive (human HeLa cervix carcinoma cells) and DT-insensitive cells (murine J774A.1 macrophage-like cell line) was analyzed by flow cytometry (Fig. 2a). Binding experiments were performed at 4 °C because endocytosis is suppressed at this low temperature due to the reduced flexibility of the membrane. In contrast to our expectations, all eGFP-labeled CRM197 proteins, i.e. in particular also the two mutants with the altered or deleted R-domain, bound to HeLa cells, albeit in flow cytometry we detected a weaker fluorescence signal presumably due to a lower-binding affinity. As expected, negative controls using ^His_^eGFP or DTA (^His_^eGFP_DTA) alone did not show an enhanced fluorescence signal for cell surface binding, proving that the observed binding is specific and not mediated by the eGFP fluorophore. We conclude from these data that foremost the R- but also the T-domain of CRM197 mediate the binding to the cell surface. Our observation that the T-domain of CRM197 also contributes to cell surface receptor binding agrees well with recently published data showing, that CRM197 has a higher-binding affinity for HB-EGF compared to a mutant carrying only the R-domain of CRM197 (Suzuki et al. 2015). To further confirm the specificity of the observed binding and the involvement of a cell surface receptor protein, we performed experiments in which the cells were pretreated with pronase to degrade the cell surface proteins prior to the incubation at 4 °C with the ^His_^eGFP-labeled DT proteins (see “Materials and methods”). Indeed, after pronase treatment none of the investigated DT mutants showed any detectable binding (Fig. 2a lower left panel), establishing that the observed binding is indeed specific and mediated by cell surface receptor proteins.

In contrast, for the murine J774A.1 cells, binding of all generated CRM197 proteins was not detectable by flow cytometry (Fig. 2a right panel). This finding is consistent with literature describing that HB-EGF of species insensitive to DT is not (or with a much lower affinity) recognized by DT (Collier 1975; Boquet and Pappenheimer 1976; Proia et al. 1979; Heagy and Neville 1981; Mitamura et al. 1995). However, when the uptake of these proteins into J774A.1 cells was investigated by incubation at 37 °C, all CRM197-based proteins were internalized while uptake of ^His_^eGFP or ^His_^eGFP_DTA was hardly detectable (Fig. 2b upper panel). To distinguish between binding of the proteins at 37 °C and their uptake into the cells, the extracellular eGFP signal was quenched with trypan blue (TB). This well-established approach was validated in a set of preliminary experiments with HeLa cells and ^His_^eGFP_CRM197 (Fig. S 4). The finding, that DT-mutants, which bind to HeLa cells are internalized into murine J774A.1 cells was unexpected, due to the fact that no binding was observed (Fig. 2a right panel). Remarkably, the mutants that do not bind to HeLa cells are also not internalized into J774A.1 cells, indicating the specificity of this uptake (compare Fig. 2a left upper panel with b upper panel). Furthermore, when the murine cells were preincubated with pronase the internalization signals were drastically reduced (compare the lower panel of Fig. 2b with upper one), indicating an interaction with a protein cell surface receptor like on DT-sensitive human HeLa cells. Disadvantageously, this binding event could not be detected using flow cytometry on J774A.1 cells. Possibly the washing steps included in this method prevent fluorescence detection of a weaker interaction, which, however, could still be sufficient to mediate internalization. Therefore, we wanted to use super-resolution fluorescence microscopy to investigate the toxin-cell interaction more directly. In these assays also milder washing steps were used due to the fact that adherent cells are used in these assays (see “Materials and methods”).

### STED super-resolution microscopy reveals CRM197 binding to murine HB-EGF

**Fig. 3:**
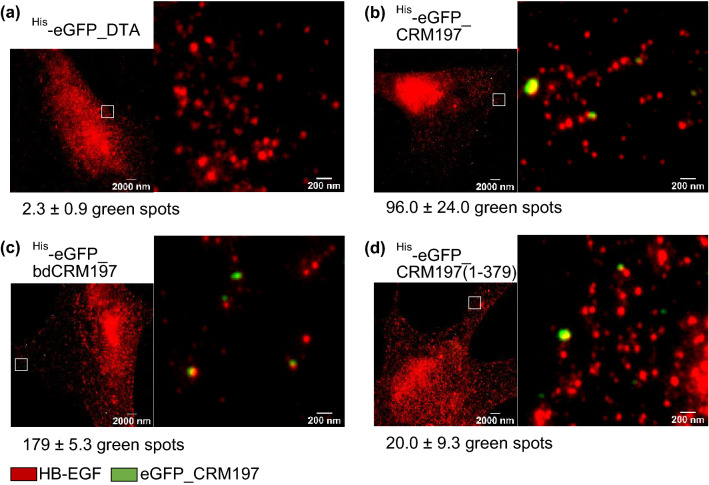
Ex vivo isolated primary rat lung fibroblasts displaying bound ^His^-eGFP_CRM197 mutants (green) in close proximity to murine HB-EGF (red) captured by STED microscopy. **a**^His^-eGFP_DTA, **b**^His^-eGFP_CRM197, **c**^His^-eGFP_bdCRM197 and **d**^His^-eGFP_CRM197(1-379) (250 nM each) were incubated on primary rat lung fibroblasts for 30 min at 4 °C. All respective mutants **b**–**d** show binding to primary rat lung fibroblasts which is specific as shown by comparison to the negative control shown in **a**. A statistical evaluation (*N* = 3 cells) of detected green spots is displayed below each subimage. Most importantly, high resolution imaging reveals significant close proximity of murine HB-EGF (red) and ^His^-eGFP_CRM197 mutants (green) as best seen in the magnified insets

While flow cytometry experiments could show differences in DT binding and uptake for different mutants and cell types we sought to obtain more mechanistic insight into the details of the binding and cellular uptake. To this end, we performed super-resolution optical microscopy (STED) experiments using dual-colors by co-staining of the toxin and binding partners (HB-EGF) or compartmental markers (e.g. endosomes, “Materials and methods”). Compared to conventional fluorescence microscopy of DT binding and uptake into murine cells which resolves structures up to 250–300 nm (Manoilov et al. 2018), the improved optical resolution of STED microscopy allows to detect close proximity of molecules down to the nanometer scale (with a lateral resolution of 35 nm, Fig. S 5) and shows sub-compartmental organization. In addition to dual-color STED imaging of CRM197 protein binding to J774A.1 cells (Fig. S 6) we also performed experiments on ex vivo isolated primary rat fibroblasts (Fig. 3) to prove that the observed effects are general and not limited to one specific cell type or species.

The cells were incubated with ^His^-eGFP_CRM197, ^His^-eGFP_bdCRM197 or ^His^-eGFP_CRM197(1-379) for 30 min at 4 °C such that the CRM197 proteins could bind to the cells but receptor-mediated internalization was absent due to the low temperature (for rat fibroblasts: Fig. 3). Moreover, we also tested for unspecific staining (Fig.  S 6, S 7, S 8) as well as for unspecific binding of ^His^-eGFP (Fig. S 7) and ^His^-eGFP_DTA to the cell surface (Fig. 3a). Quantitative comparison showed that binding was indeed specific (10 to 100-fold increase in localizations, depending on the affinity of the respective mutant). All tested CRM197 mutants with functional T-domain showed specific binding to the cellular membrane (Fig. 3b–d) and are closely associated with the murine HB-EGF. Interestingly, these STED microscopic findings are perfectly in accordance with the observations made on J774A.1 cells in the flow cytometry uptake studies (Fig. 2b upper panel). However, they are contrary to the flow cytometric binding studies on J774A.1 cells (Fig. 2a right panel). As mentioned above we interpret the difference in the observed binding behavior to the different experimental washing procedures. This observation could help to explain the reported controversy in the field and leads to the conclusion that only highly sensitive methods combined with less harsh washing steps can unveil this interaction. In particular, STED microscopy establishes that ^His^-eGFP_bdCRM197 which has previously been identified to have a reduced affinity for HB-EGF, as well as ^His_^eGFP_CRM197(1-379), lacking the complete putative receptor-binding domain, both co-localize with HB-EGF in mouse (Fig. S 6) and rat cells (Fig. 3). Notably, the eGFP-signals are directly associated with the HB-EGF, while the spacing between two different detected HB-EGF signals is comparatively large. Therefore, the co-localization cannot be explained by statistical coincidence but necessitates a specific interaction of the two proteins, which can be either direct or indirect. Interaction of ^His^-eGFP_CRM197(1-379) with murine HB-EGF shows that CRM197 carries receptor-recognizing sequences within the first 379 amino acids. Combined with the observation that ^His_^eGFP_DTA shows negligible co-localization with the HB-EGF the interaction has to occur within the T-domain (amino acids 194-379). Moreover, quantitative analysis of co-localization events from STED imaging allows at least a qualitative comparison of the binding affinities of the different mutants. We observe 3–5 times fewer binding events for ^His_^eGFP_CRM197(1-379) as compared to full length CRM197 on all cell-types (Fig. S 6, S 7, S 8), which is in agreement with the data obtained for the HeLa cells in flow cytometry (Fig. 2a, left panel). Moreover, using STED super-resolution microscopy, it was validated that also Hela cells which carry the human HB-EGF interact with ^His^-eGFP_CRM197, ^His^-eGFP_bdCRM197 and ^His^-eGFP_CRM197(1-379) under same conditions in a comparable manner (Fig. S 8). Taken together these results show that the different amino acid sequences of the murine HB-EGF reduce the binding affinity of CRM197 compared to sensitive mammals, but do not fully block binding of CRM197 to the murine HB-EGF. Hence, the next step in our study was the evaluation of the cellular uptake of CRM197 upon receptor binding into murine cells and primary rat fibroblasts.

### DT-resistant murine cells internalize CRM197 via the early endosomal pathway

**Fig. 4:**
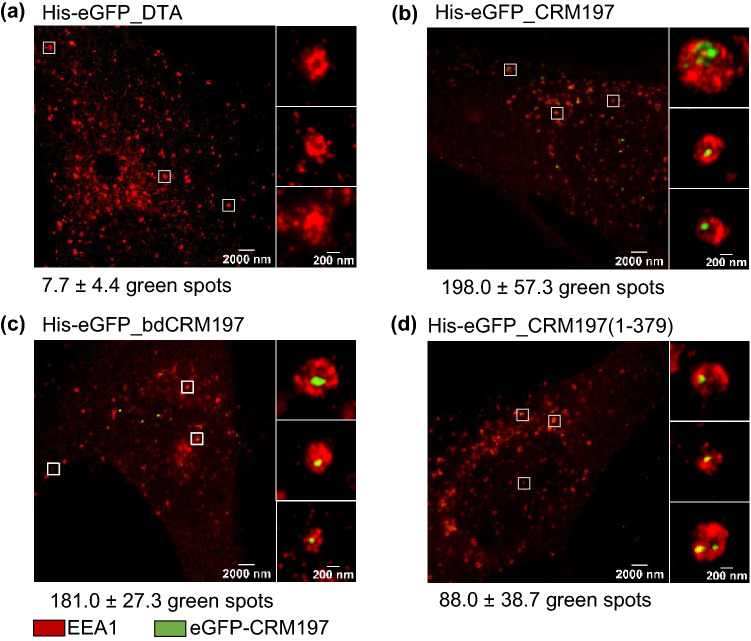
Primary rat lung fibroblasts showing early endosomal antigen 1 (EEA1) (red) and His-eGFP_CRM197 mutants (green) captured with STED super-resolution microscopy. Incubation of **a**^His^-eGFP_DTA, **b**^His^-eGFP_CRM197, **c**^His^-eGFP_bdCRM197 as well as **d**^His^-eGFP_CRM197(1-379) for 30 min at 37 °C exhibit internalization of ^His^-eGFP_CRM197 mutants (green) into early endosomes (red). Magnified areas (regions indicated by white squares) show that the CRM197 protein is contained within the endosomal compartments (**b**–**d**) while the compartments in the negative control (**a**) show no detectable fluorescence signal from the labelled toxin protein. A statistical evaluation (*N* = 3 cells) of detected green spots is displayed below each subimage

To test for cellular uptake of CRM197 into murine cells, J774A.1 macrophages (Fig. S 9) were incubated with ^His^-eGFP labeled proteins at 37 °C for 30 min, fixated, immunostained and imaged using dual-color STED microscopy. Again negative controls ruled out unspecific immunostaining and excluded the possibility of unspecific non-receptor-mediated internalization or other passive transports of ^His^-eGFP (Fig. S 9, S 10). Co-localization with the early endosomal antigen 1 (EEA1) reveals that ^His^-eGFP_CRM197, ^His^-eGFP_bdCRM197 as well as ^His^-eGFP_CRM197(1-379) were internalized by the endosomal pathway into the murine cells (Fig. S 9). However, since J774A.1 cells derived from a monocyte-macrophage tumor, these cells may exhibit a preference for engulfing foreign substances. Therefore, we validated the internalization data obtained from J774A.1 with another non-monocyte-macrophage cell type, namely primary rat lung fibroblasts. The obtained results show that all investigated DT derived proteins containing the amino acids 194-379 interact with the murine HB-EGF (Fig. 3), which leads to active receptor-mediated endocytosis (Fig. 4). These findings suggest that the previously described receptor-binding domain might not be the only structure involved in receptor-mediated cellular uptake. This surprising finding could be explained by a receptor-recognizing element located in the T-domain of CRM197. However, the involvement of co-receptors like DRAP27/CD9 (Iwamoto et al. 1994) or caveolin-1 (Lin et al. 2016) is also possible. Earlier publications have shown that CD9 from sensitive species can serve as a co-receptor for DT (Iwamoto et al. 1994; Cha et al. 2000). However, mouse CD9 did not enhance the cellular binding when it is co-expressed with monkey (DT-sensitive) HB-EGF (Cha et al. 2000). Therefore, it remains unclear if the murine CD9 does not interact with DT or does not bind the monkey HB-EGF homologue. Furthermore, also caveolin-1 could serve as a possible co-receptor. A caveolin-binding motive within the T-domain of DT or CRM197 has recently been found and it was shown that a short fragment of DT (amino acids 270-326) directly interacts with caveolin-1 (Lin et al. 2016).

While we do observe cellular uptake of toxins into the putatively toxin insensitive cell lines, we confirmed that the murine cells used in this study show very little to no sensitivity to even a high dosage of DT (Supplements & Fig. S 1). Notably, we also confirmed and extended earlier studies showing that the murine eEF2 can be ADP-ribosylated by DTA when it reaches the cytosol of mouse and rat cells (Fig. S 1, S 2). Therefore, we conclude that the resistance of murine cells to DT is associated with a defect in the later stages of the internalization processes. A likely step could be the translocation of DTA from endosomal vesicles into the cytosol. An explanation for the observed results and the majority of published data is that the binding affinity of DT for the murine HB-EGF is reduced but still sufficient for cellular internalization. In the early endosomes, however, this interaction might be too weak for sufficient translocation of DTA across the endosomal membrane and into the cytosol. In this model, a strong DT-receptor interaction would be needed to insert the T-domain into the endosomal membrane and thereby initiate the translocation step. Further detailed investigations, also with diffraction-unlimited STED super-resolution optical microscopy are, therefore, needed to unveil the actual molecular mechanisms underlying this mode of action.

## Electronic supplementary material

Below is the link to the electronic supplementary material.

Supplementary file1 (PDF 1511 kb)
