# Supplementary material for: Super-resolution microscopy unveils transmembrane domain-mediated internalization of cross-reacting material 197 into diphtheria toxin-resistant mouse J774A.1 cells and primary rat fibroblasts in vitro
Source: Arch Toxicol. 2020 Apr 8;94(5):1753–61. doi: 10.1007/s00204-020-02731-4 (PMC7261736; doi:10.1007/s00204-020-02731-4)
Supplement: Supplementary file 1 — Supplementary file1 (PDF 1511 kb) [file 204_2020_2731_MOESM1_ESM.pdf]

Super-resolution microscopy unveils transmembrane domain-mediated internalization of cross-reacting material 197 into diphtheria toxin-resistant mouse J774A.1 cells and primary rat fibroblasts *in vitro*

Maximilian Fellermann[1,#], Fanny Wondany[2,#], Stefan Carle[1], Julia Nemeth[3], Tanmay Sadhanasatish [2], Manfred Frick[3], Holger Barth[1,*], Jens Michaelis[2,*]

[1] Institute of Pharmacology and Toxicology – Ulm University Medical Center, Albert-Einstein-Allee 11, 89081 Ulm, Germany

[2] Institute of Biophysics - Ulm University, Albert-Einstein-Allee 11, 89081 Ulm, Germany

[3] Institute of General Physiology - Ulm University, Albert-Einstein-Allee 11, 89081 Ulm, Germany

[#] These authors contributed equally.

[*] Correspondence: holger.barth@uni-ulm.de, jens.michaelis@uni-ulm.de

Materials

Elastase was purchased from Elastin Products Co (USA). Trypsin and DNase from Sigma-Aldrich (USA) were used. For the rat fibroblast isolation FCS from HyClone, GE Healthcare Life Sciences, (GBR) was used. CD45 MicroBeads and CD90.1 MicroBeads were from Miltenyi Biotec (GER). MucilAir from Epithelix (SUI) with Gentamicin from Life Technologies (USA) was used. Dulbecco's Modified Eagle's medium (DMEM), Minimum Essential Medium Eagle (MEM), fetal calf serum (FCS), penicillin-streptomycin, sodium pyruvate, L-glutamine and non-essential amino acids were purchased from GIBCO® life technologies (USA). Cell culture dishes and 24-well plates form TPP® Techno Plastic Products AG (SUI) were used. Ibidi GmbH (GER) provided the glass bottom 8-well µ-slides. PfuUltra II HS DNA Polymerase for site-directed mutagenesis was from Agilent Technologies Inc. (USA). The In-Fusion® HD form Cloning (USA) was used. The primers were synthesized by biomers.net GmbH (GER). dNTPs and the *Escherichia coli* stain Shuffle® T7 Express from New England Biolabs (USA) were used. IPTG form AppliChem GmbH (GER) was used. For purification the ÄKTA Explorer FPLC system from GE Healthcare Life Sciences (USA) was used with a Protino® Ni-NTA Column 1 mL from Macherey-Nagel GmbH & Co.KG (GER). Vivaspin 20® centrifugal concentrators (30 kDa MWCO) were purchased from Sigma-Aldrich Chemie GmbH (USA). The FACSCelesta™ from BD-Bioscience (USA) was used in combination with flowing software 2.5.1 created by Perttu Terho (Turku Centre for Biotechnology (FIN)). PFA solution (32 %, EM grade) was purchased from Electron Microscopy Science (USA). BSA (heat shock fraction, protease free, fatty acid free, essentially globulin free, pH 7, ≥98 %), 2,2′-Thiodiethanol (≥ 99.0 %), goat anti-rabbit IgG conjugated with Atto647N (1 mg/mL) and goat anti-mouse IgG conjugated with Atto647N (1 mg/mL) was obtained from Sigma-Aldrich corp. (USA). TritonX-100 was received from Merck KGaA (GER). GFP-Booster was acquired by purchase from Chromotek (GER). Mouse HB-EGF antibody (H-1: sc-365182, 200 µg/mL) was obtained from Santa Cruz Biotechnology (USA). Rabbit anti-EEA1 (1 mg/mL) was purchased from Thermo Scientific (USA). The native unnicked DT was from Calbiochem Merck Millipore (USA). MTS-CellTiter 96® AQueous One Solution Cell Proliferation Assay from Promega GmbH (USA) was used at the microplate reader TriStar² LB942 from Berthold Technologies GmbH & Co.KG (GER) The protease inhibitor cOmplete™ was

purchased from Roche Diagnostics GmbH (SUI). 6-biotin-17-NAD^+ from Trevigen (USA), nitrocellulose blotting membrane from GE Healthcare Life Sciences (USA) were used while the streptavidin-peroxidase (500 u/mL) and the Tween®20 were from Sigma-Aldrich corp. (USA). The Glyceraldehyde 3-phosphate dehydrogenase (GAPDH) antibody (G-9: sc-365062, 200 µg/mL), mouse anti-rabbit IgG-HRP (sc-2357) and the mouse IgG_K binding protein-horseradish peroxidase (sc-516102, 400 µg/mL) was from Santa Cruz Biotechnology (USA). Skim milk powder was purchased from AppliChem GmbH (GER). The Immobilon™ Western Chemiluminescent HRP Substrate was from Millipore corp. (USA). The medical X-Ray Film CX-BL+ form AGFA Health Care N.V. (BEL) were used. Diphtheria toxin antibody (7F2) was purchased from GeneTex, while the rabbit anti-GFP antibody (ab290) from Abcam was used.

The proteins ^{His}-eGFP, ^{His}-eGFP_DTA, ^{His}-eGFP_CRM197, ^{His}-eGFP_bdCRM197, ^{His}-eGFP_CRM197(1-379), ^{His}-DTA, were recombinantly produced in *Escherichia coli*.

The basic plasmid pTRC99A: myc-DT_{E148S} was kindly provided by Dr. Emmanuel Lemichez (Paris, FRA). The plasmid containing the DNA for the eGFP was kindly provided by Dr. Joachim Orth/Dr. Klaus Aktories (Freiburg, GER). The plasmid pET15b:^{His}-DTA, the recombinantly produced PA_{63} and untagged DTA were kindly provided by Dr. R. John Collier (Boston, USA).

Supplementary methods

Molecular cloning of ^{His}-eGFP-labeled proteins

For molecular cloning, the basic plasmid pTRC99A:myc-DT_{E148S} was kindly provided by Dr. Emmanuel Lemichez (Paris, FRA). pTRC99A:^{His}-CRM197 was created using site-directed mutagenesis and overhang PCR with traditional cloning techniques. The plasmid-DNA for the eGFP sequence was kindly provided by Dr. Joachim Orth/Dr. Klaus Aktories (Freiburg, GER). It was amplified and inserted into pTRC99A:^{His}-CRM197 using the In-Fusion® cloning kit. The resulting pTRC99A:^{His}-eGFP_CRM197 plasmid contains a GSG-linker between eGFP and CRM197. Additionally, the amplified eGFP was inserted into the pTRC99A backbone using traditional cloning techniques resulting in pTRC99A:^{His}-eGFP. For pTRC99A:^{His}-eGFP_DTA the insert of eGFP was amplified and fused with DTA using traditional cloning techniques.

Using further site-directed mutagenesis pTRC99A:^{His}-eGFP_bdCRM197 (insertion of L390F, S525F mutations) and pTRC99A:^{His}-eGFP_CRM197(1-379) (Insertion of two stop codons at position 380 and 381) were generated. All plasmids were sequenced to validate the correctness of the sequence.

Recombinant production of His-tagged proteins

The plasmids were transformed into competent *Escherichia coli* Shuffle® T7 Express. A single colony was picked cultured for one day at 30 °C and 180 rpm in 5 mL LB-medium containing ampicillin (LB-Amp: 10 g/L tryptone, 5 g/L yeast extract, 10 g/L NaCl, 100 mg/mL ampicillin). A 200 mL overnight culture in fresh LB-Amp was inoculated with the culture and incubated at 30 °C and 180 rpm in an Erlenmeyer flask. The next day, 4x1 L of LB-Amp in 2 L Erlenmeyer flasks were inoculated with 35 mL of overnight culture per flask and further incubated at 30 °C and 180 rpm. Upon an OD_{600} of 0.4-0.7 was reached the expression was induced using 0.5 mM IPTG and temperature was decreased to 16 °C for overnight incubation at 180 rpm. Cells were harvested by centrifugation at 5500 rcf, 4 °C for 10 min. Equilibration buffer (50 mM NaH_2PO_4, 300 mM NaCl, 20 mM imidazole, pH 8.0) was used to resuspend the

cell pellets and freeze them. Subsequently, pellets were thawed, 1 mM PMSF was added and sonicated on ice (10x30 s pulses with an amplitude of ~ 40 % and intermediate pauses of 30 s). Centrifuged at 13000 rcf for 30 min and 4 °C and filtration through 0.45 µm and 0.2 µm syringe filters was used to remove cells and membrane fragments. For purification an ÄKTA® FPLC system was used with a 1 mL Ni-NTA column. Elution of the proteins was performed with elution buffer (50 mM NaH_2PO_4, 300 mM NaCl, 250 mM imidazole, pH 8.0). The fractions containing the protein of interest were pooled and buffer exchange to PBS was performed using Vivaspin 20® centrifugal concentrators with 30 kDa MWCO (refilled three times with 20 mL PBS). The protein-solutions were stored at -80 °C. The concentrations were determined using SDS-PAGE and a BSA standard curve.

Sample preparation and immunostaining for microscopy experiments

Primary rat lung fibroblasts ($3.0 \cdot 10^5$/well), murine J774A.1 cells ($2.5 \cdot 10^4$/well) and HeLa cells ($1.8 \cdot 10^4$/well) were seeded in 8-Well ibidi µ-Slide. The cells were incubated with the respective proteins (250 nM) at the indicated temperature and time in FCS-free medium. Cells were fixated with 3.2% (v/v) paraformaldehyde (PFA) for 20 min at room temperature (RT). Three washing steps with PBS were applied to remove the remaining PFA. Cell permeabilization and blocking of unspecific binding sites was accomplished by 2 h incubation at RT with 3% (w/v) BSA and 0.3% (v/v) TritonX-100 dissolved in PBS. The supernatant was removed and 1 µg/mL GFP-booster nanobody conjugated with Atto594, mouse anti-HB-EGF antibody or rabbit anti-EEA1 antibody was incubated overnight at 4 °C dissolved in 0.3% (w/v) BSA and 0.03% TritonX-100 (v/v). Thereafter, three washing steps with PBS removed unbound primary antibody. Subsequent second antibody incubation was performed for 1 h at RT with 1 µg/mL secondary antibody – i.e. with goat anti-rabbit antibody conjugated with Atto647N or goat anti-mouse antibody conjugated with Atto647N – dissolved in in 0.3% (w/v) BSA and 0.03% TritonX-100. Three washing steps with PBS removed unbound antibody. Before imaging, PBS was exchanged for 2,2'-thiodiethanol (97 % solution in PBS, pH 7.5).

Cell viability assay

10^6 murine macrophage-like J774A.1 cells were seeded per 96-well plate and grown for 1 day. 10^7 isolated primary rat fibroblasts were seeded per 96-well plate and grown for six to seven days. Subsequently the cells were treated with DT (250 nM), recombinant PA_{63} (4 nM) in combination with ^{His}-DTA (40 nM), PA_{63} (4 nM) alone, ^{His}-DTA alone or left untreated (negative control (NC)). After one and two days 10 µL MTS were added and the cells were incubated for 1 h at 37 °C The relative absorbance at 492 nm was measured (reflecting the cell viability/proliferation). The means of one triplicate (n=3) ± standard deviations (SD) were normalized to the negative control.

In vitro ADP-ribosylation assay in cell lysate

Cell lysates of mouse J774A.1 or primary rat fibroblast were prepared in ADP-ribosylation buffer (20 mM Tris-HCl, 1 mM EDTA, 1 mM DTT, 5 mM $MgCl_2$, cOmplete™ (1:50, freshly added), pH 7.5).

Equal amounts of the lysates were incubated with an excess of 6-biotin-17-NAD^+ (10 µM) and either DTA (5 pmol) or DT (5 pmol) at 37 °C for 30 min. The reactions were started and stopped at the same time. Afterwards, Western blotting and detection of the biotinylated eEF2 with streptavidin-peroxidase (1:5000) was performed. GAPDH was

detected to confirm comparable protein loading. The GAPDH-antibody (1:2000) was used in combination with mouse IgG_K binding protein-horseradish peroxidase (1:2500) conjugate.

Post ADP-ribosylation assays to investigate the ADP-ribosylation status of eEF2 in intact cells

Primary rat lung fibroblasts ($4 \cdot 10^5$/well) and mouse J774A.1 cells ($6 \cdot 10^4$/well) were seeded in a 24-well plate. The cells were treated with DT (250 nM), PA_{63} (4 nM) in combination with ^{His}-DTA (40 nM) or left untreated (NC). For comparison with DT-sensitive cells HeLa cells ($4 \cdot 10^4$/well) were treated with 0.01 nM DT. After 24 h the cells were washed twice with PBS (137 mM NaCl, 2.7 mM KCl 8 mM Na_2HPO_4 and 1.8 mM KH_2PO_4, pH 7.4). Subsequently, the cells were lysed by freezing and thawing on ice in ADP-ribosylation buffer (see 4.2.5). On ice, DTA (5 pmol) and 6-biotin-17-NAD^+ (10 µM) were added and the reactions at 37 °C were started and stopped at the same time after 30 min. Afterwards, Western blotting and detection of the biotinylated eEF2 with streptavidin-peroxidase (1:5000) was performed. Note that a weak signal indicates that most eEF2 was already ADP-ribosylated by the toxin in the intact cells prior to lysis. GAPDH was detected to confirm comparable protein loading. The GAPDH-antibody (1:2000) was used in combination with mouse IgG_K binding protein-horseradish peroxidase (1:2500) conjugate.

SDS-PAGE and Western blotting

Size-dependent protein-separation was obtained in SDS-PAGE. Using a semi-dry blot chamber, the proteins were transferred to a nitrocellulose membrane. Unspecific binding was blocked by incubation in 5 % skim milk powder in PBS-T (137 mM NaCl, 2.7 mM KCl 8 mM Na_2HPO_4, 1.8 mM KH_2PO_4, 0.1 % Tween®20, pH 7.4) for 1 h at room temperature (RT). Afterwards, the membrane was incubated with the respective primary antibody or streptavidin-peroxidase conjugate. Subsequent to washing (three times with PBS-T for 5 min at RT on an orbital shaker) a peroxidase conjugated secondary antibody was added and incubated for 1 h RT. Three further washing steps were performed, and the peroxidase marked sites were detected with chemiluminescent HRP substrate and medical X-ray films.

Supplementary results: Resistance of mouse and rat cells towards DT depends on insufficient transport of the enzyme subunit DTA into the cytosol

In a first set of experiments, we confirmed that the murine cells used in this study are indeed resistant towards DT. To this end, either the mouse J774A.1 macrophage-like cell line or *ex vivo* isolated primary rat fibroblasts were challenged with high concentrations of DT and cell viability was determined after one and two days. There was no detectable decrease in the rat fibroblast and only a negligible decrease in the J774A.1 cells regarding the amount of viable cells after treatment with DT, confirming the resistance towards the cytotoxic effects of DT (Fig. S 1 a). However, if the isolated enzyme domain of DT (^{His}-DTA) is delivered into the cytosol of these murine and rat cells via an artificial transport system, the viability of both cell types was strongly reduced. For delivery of ^{His}-DTA the well-established anthrax toxin-based transport system (PA_{63}) (Blanke et al. 2002) was used. Noteworthy, neither ^{His}-DTA nor PA_{63} alone induced such cytotoxic effects. The results clearly show that the enzyme domain of DT, DTA, is cytotoxic to mouse and rat cells when it is delivered into their cytosol. This was also confirmed by analyzing the ADP-ribosylation status of eEF2 in these cells (Fig. S 1 b).

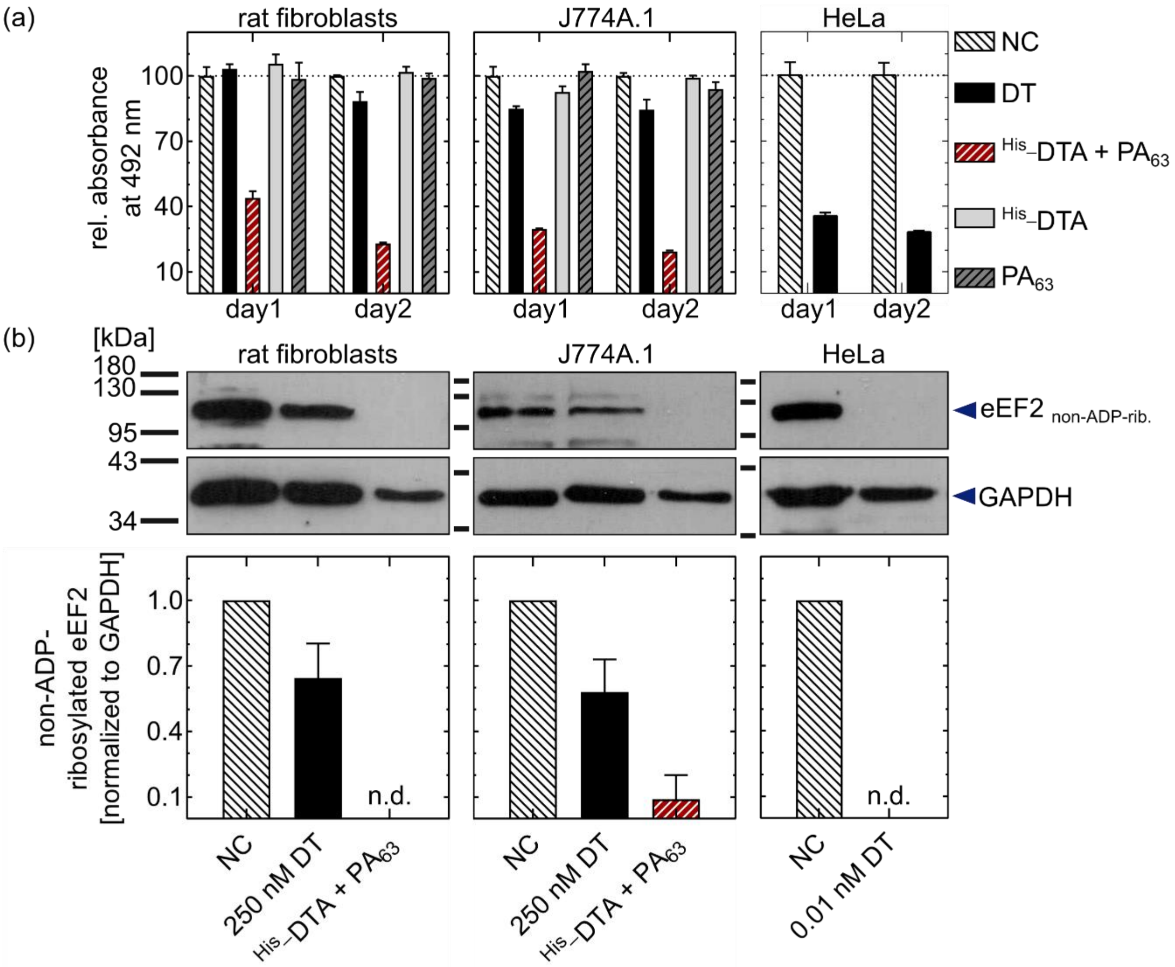

Fig. S 1

Effect of high DT concentrations and a combination of ^{His}-DTA + PA_{63} on mouse J774A.1 macrophages and isolated primary rat fibroblasts. (a) The cells were treated with either DT (250 nM), or with ^{His}-DTA (40 nM) in combination with PA_{63} (4 nM), or with either ^{His}-DTA (40 nM) or with PA_{63} (4 nM) alone. For comparison DT-sensitive HeLa cells were treated with 0.01 nM DT. After one and two days MTS was added and the relative absorbance at 492 nm was measured (reflecting the cell viability/proliferation). NC (negative control), i.e. cells were left untreated. The values are given as mean of three measurements ± standard deviation (SD). (b) J774A.1 and primary rat fibroblast were treated with DT (250 nM) or a combination of ^{His}-DTA (40 nM) with PA_{63} (4 nM) for 24 h. HeLa cells were treated with 0.01 nM DT. Then, cells were lysed and lysate protein incubated *in vitro* with biotin-NAD and fresh DTA. The non-ADP-ribosylated (i.e. biotin-labeled) eEF2 was analyzed by Western blotting (upper panel). Note: a weak signal indicates that eEF2 was already ADP-ribosylated in the intact cells during incubation with the toxin. Glyceraldehyde 3-phosphate dehydrogenase (GAPDH) was detected to control protein loading. Lower panel: The signals of three Western blots were quantified if detectable (n.d.= not detectable). The signal for the non-ADP-ribosylated eEF2 was normalized to the loading control (GAPDH). The values are given as mean ± standard deviation.

High DT concentrations only show minimal effects on the ADP-ribosylation of murine cells while minimal portions of DT on toxin-sensitive human cervix carcinoma cells (HeLa) show much stronger effects. Moreover, further experiments show that murine eEF2 serves as a substrate for DT and DTA, since eEF2 is ADP-ribosylated in mouse and rat cell lysates (Fig. S 2). In conclusion, the results confirm and extend earlier findings (Goor et al. 1967; Moehring and Moehring 1968) that the observed DT-resistance of mouse and rat cells results from insufficient transport of DTA into the cytosol. While earlier reports showed that mouse and rat eEF2 is a substrate for DTA in cell lysates *in vitro*, we demonstrated that DTA efficiently ADP-ribosylates eEF2 in murine cells as soon as DTA can be delivered into their cytosol by artificial transport systems.

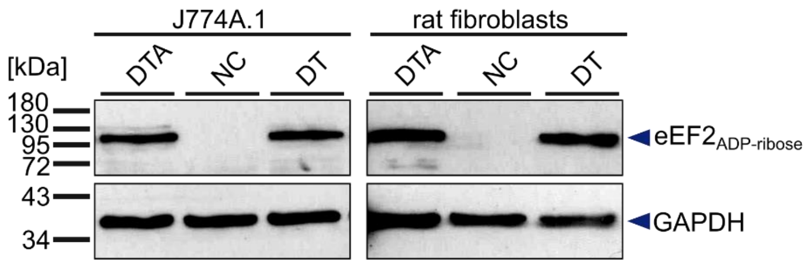

Fig. S 2

Murine eEF2 serves as substrate for the DTA-catalyzed ADP-ribosylation *in vitro*. Lysates of mouse J774A.1 cells or primary rat fibroblasts were incubated with an excess of biotin-NAD and either DTA or DT at 37 °C for 30 min. Afterwards, the ADP-ribosylation status of eEF2 was analyzed (indirect biotinylation) by Western blotting and detection with streptavidin-peroxidase. Note: A strong signal indicates strong ADP-ribosylation. Glyceraldehyde 3-phosphate dehydrogenase (GAPDH) was detected to confirm comparable protein loading.

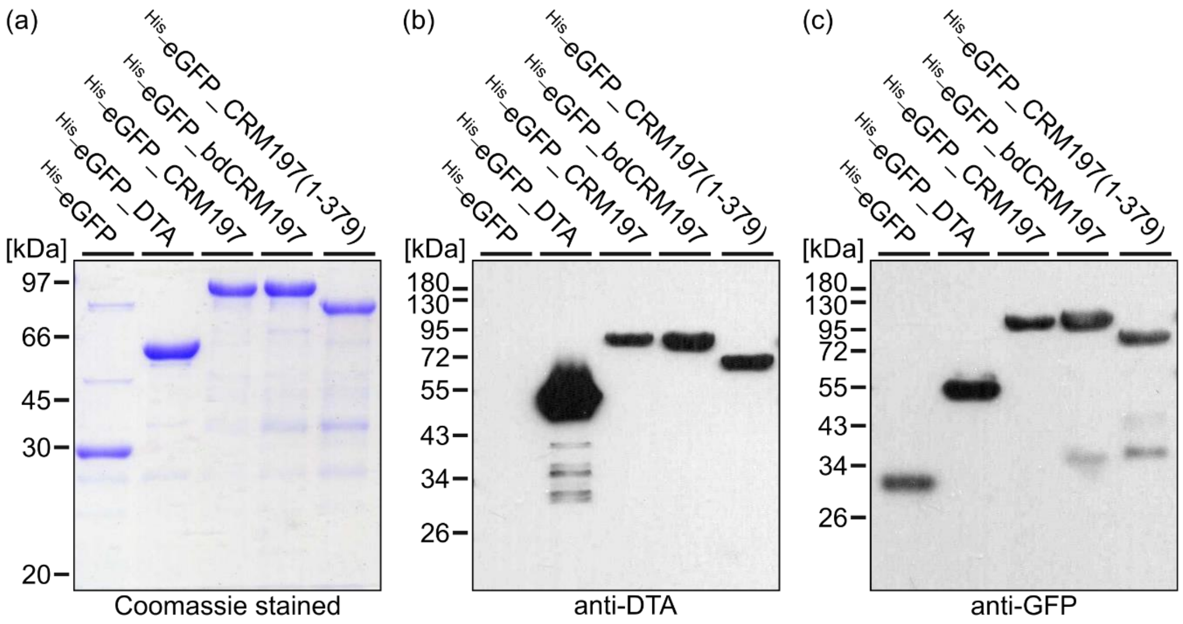

Fig. S 3

Coomassie brilliant blue R250 stained SDS-gel and Western blot detection of DTA and GFP to confirm the purity and identity of the purified eGFP-labelled proteins. Therefore, 2 µg of each eGFP-labeled protein (^{His}eGFP, ^{His}eGFP_DTA, ^{His}eGFP_CRM197, ^{His}eGFP_bdCRM197, ^{His}eGFP_CRM197(1-379)) were loaded on three separate SDS-gels. (a) One gel was stained with a Coomassie brilliant blue R250 solution (2.5 % (m/v) Coomassie brilliant Blue R250, 10 % (v/v) methanol, 45 % (v/v) glacial acetic acid). Afterwards, the background was destained (10 % (v/v) methanol, 45 % (v/v) glacial acetic acid). (b, c) The two other gels were semi-dry blotted on a nitrocellulose membrane. After blocking in a 5 % skim milk powder solution, DTA and eGFP were detected. (b) For the detection of DTA the diphtheria toxin antibody was diluted 1:2000 in PBS and used beforehand incubation with mouse IgG$_{K}$ binding protein-horseradish peroxidase (1:2500) conjugate. (c) eGFP was detected with rabbit anti-GFP (1:10000) and mouse anti-rabbit IgG-HRP (1:2500).

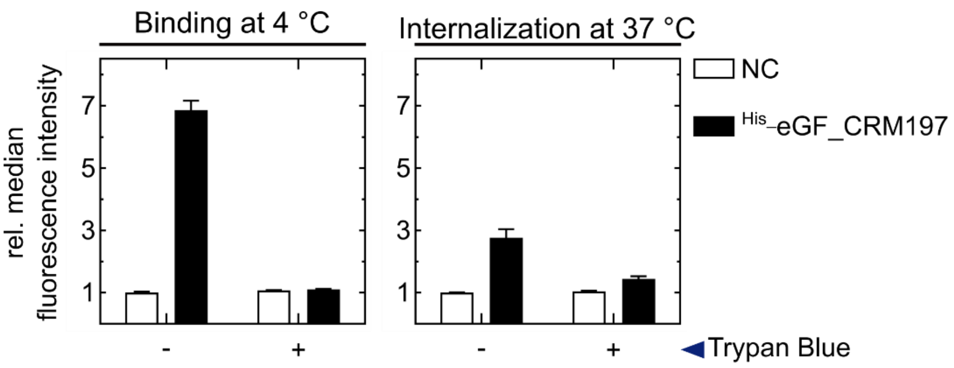

Fig. S 4

Trypan Blue application quenches the extracellular (membrane-associated) eGFP-signals. HeLa cells were detached by incubation with 25 mM EDTA (20 min at 37 °C). Subsequent to washing, the cells were treated with His–eGFP_CRM197 (250 nM) for 20 min at 4 °C, or alternatively, for 30 min at 37 °C. Afterwards, the cells were washed twice with ice-cold PBS and Trypan Blue was added to a final concentration of 50 ng/µL. Subsequently, the cells were analyzed by flow cytometry. The column-diagrams show the relative median fluorescence intensity at 488 nm excitation (gated for the main population and normalized to the NC).

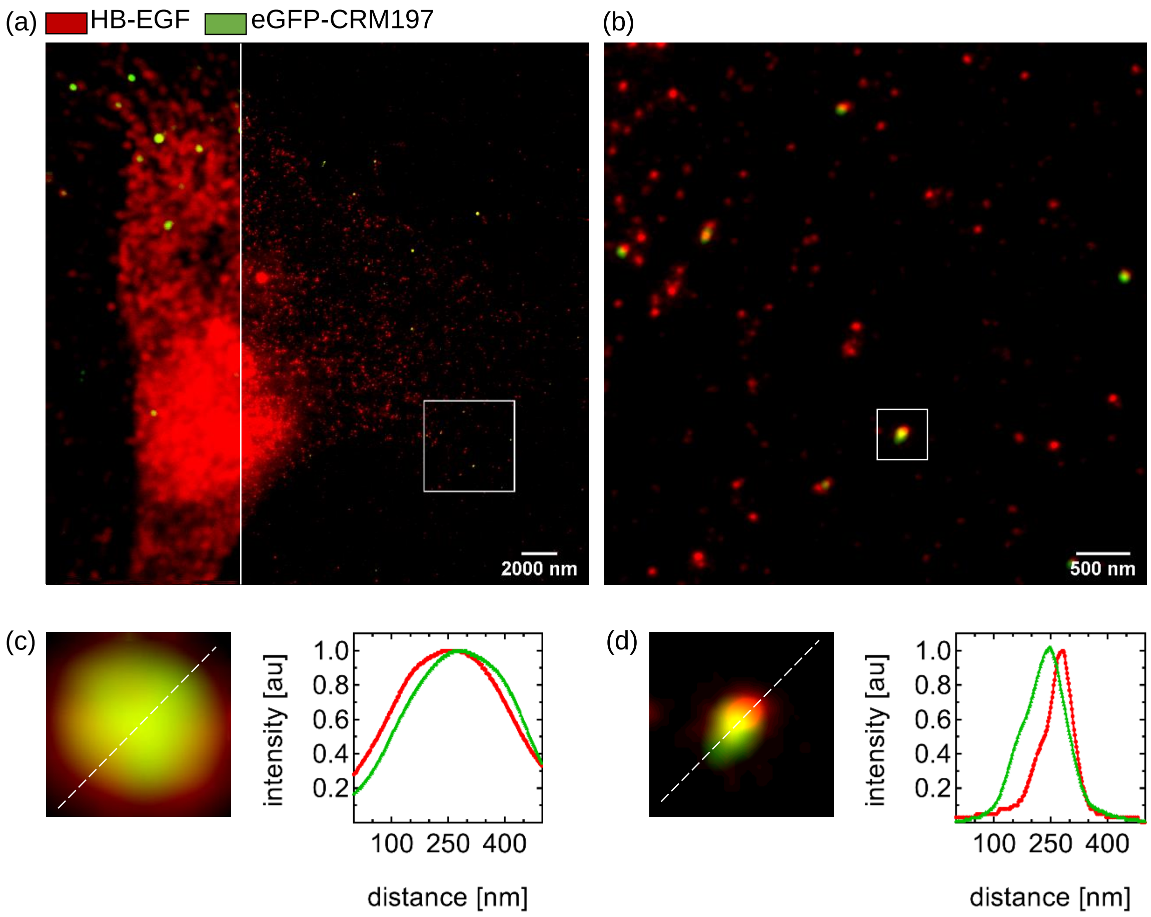

Fig. S 5

Exemplary dual-color diffraction-limited confocal and diffraction-unlimited super-resolution STED imaging of immunostained HB-EGF and ^{His}-eGFP_CRM197 in fixated rat fibroblast. (a) shows a single rat fibroblast in a 30 x 30 µm field of view highlighting the improvement in resolution between diffraction limited confocal resolution (left third) and STED super-resolution (right two thirds). Square indicates location of zoomed area displayed in (b) allowing detailed view of super-resolved structures. An area with a reduced number of HB-EGF signals was chosen to show the specificity of this co-localization and excluding the statistical coincidence. Square in (b) indicates the area of a distinct ^{His}-eGFP_CRM197/HB-EGF spot, which is compared in (c) and (d) regarding diffraction-limited (confocal, c) and diffraction-unlimited (STED, d) resolution. Dashed lines indicate position and direction of each cross section in (c) and (d), quantitatively emphasizing the enhancement in resolution. Overlay of green (^{His}-eGFP_CRM197 mutants) and red (HB-EGF) signals result in yellow colors for positions where both dyes are detected.

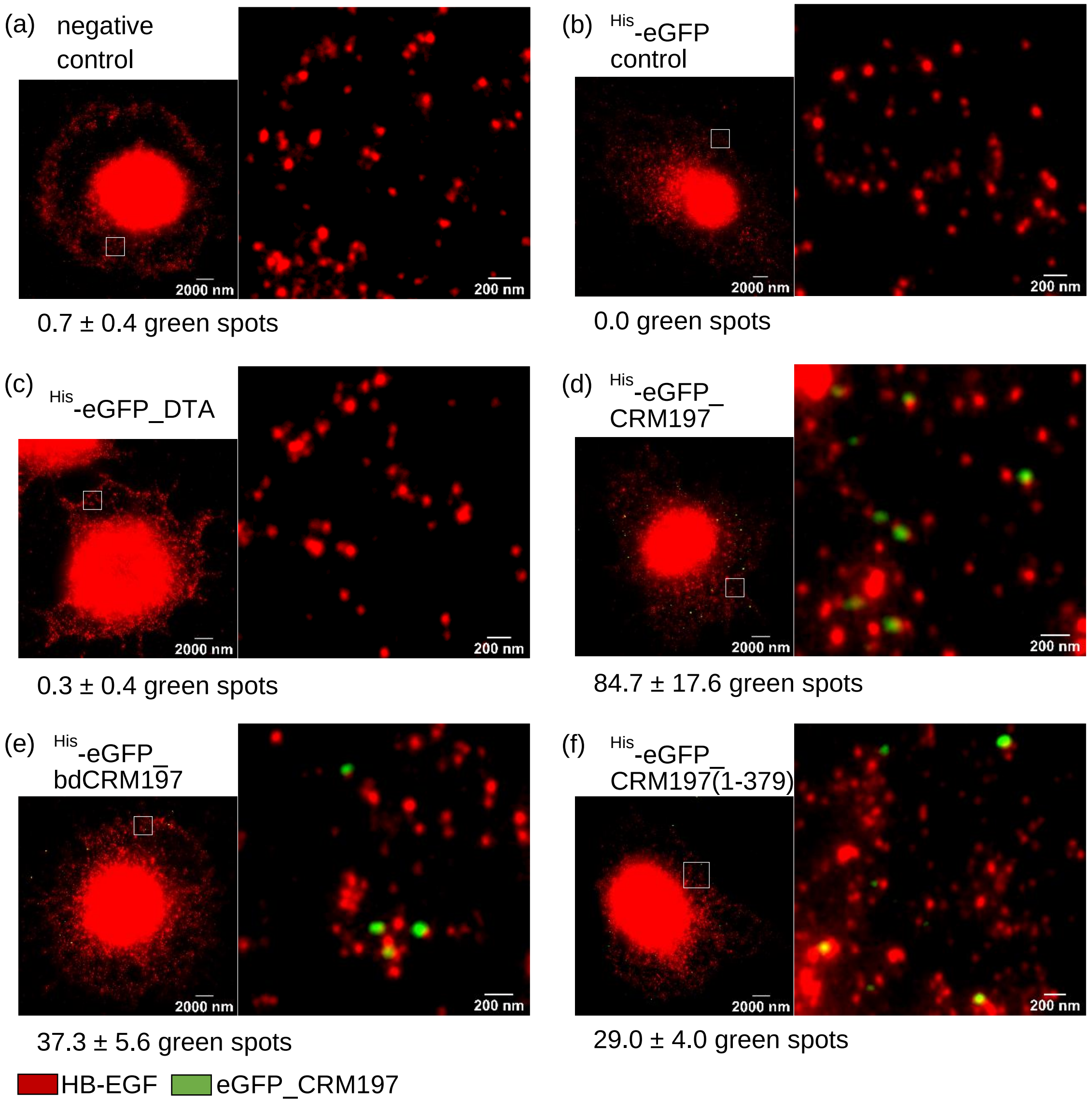

Fig. S 6

Mouse J774A.1 macrophage cell-line displaying bound ^{His}-eGFP_CRM197 mutants in close proximity to murine HB-EGF captured by STED microscopy. (b) ^{His}-eGFP, (c) ^{His}-eGFP_DTA, (d) ^{His}-eGFP_CRM197, (e) ^{His}-eGFP_bdCRM197 as well as (f) ^{His}-eGFP_CRM197(1-379) (250 nM each) were incubated on mouse J774A.1 macrophage cells for 30 min at 4 °C. Negative control, ^{His}-eGFP control and ^{His}-eGFP_DTA (a - c) do neither show unspecific binding of ^{His}-eGFP or ^{His}-eGFP_DTA to cellular surface nor staining of unspecific structures after incubation with GFP-Booster. All respective mutants (d – f) show binding to mouse J774A.1 macrophage cells. Moreover, zooms of distinct ^{His}-eGFP_CRM197 areas (white squares) reveal significant close proximity of murine HB-EGF (red) and ^{His}-eGFP_CRM197 mutants (green). A statistical evaluation (N = 3 cells) of detected green spots is displayed below each subimage.

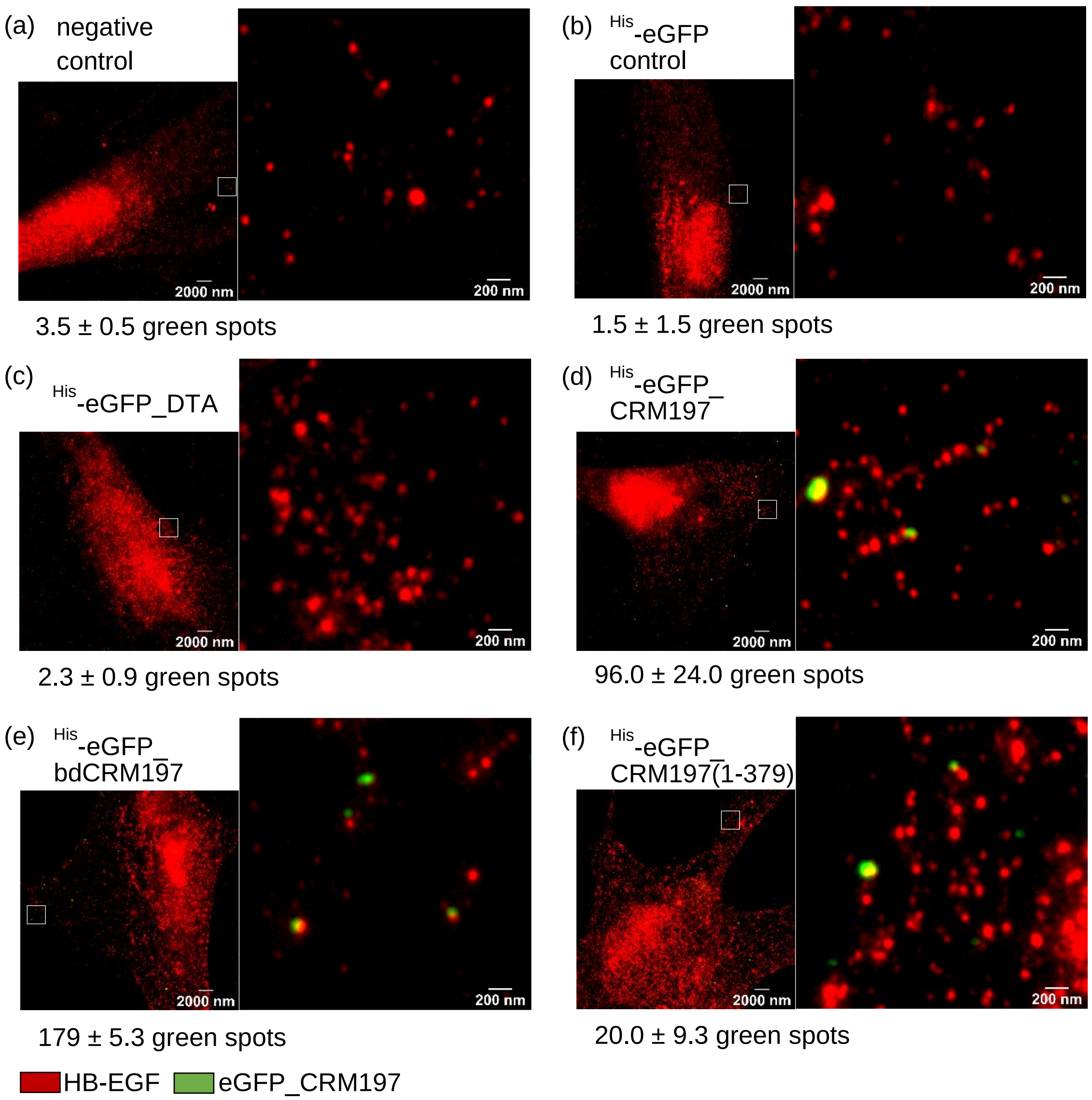

Fig. S 7

Primary rat lung fibroblasts displaying bound ^{His}-eGFP_CRM197 mutants in close proximity to murine HB-EGF captured by STED microscopy. (b) ^{His}-eGFP, (c) ^{His}-eGFP_DTA, (d) ^{His}-eGFP_CRM197, (e) ^{His}-eGFP_bdCRM197 as well as (f) ^{His}-eGFP_CRM197(1-379) (250 nM each) were incubated on primary rat lung fibroblasts for 30 min at 4 °C. Negative control, ^{His}-eGFP control and ^{His}-eGFP_DTA (a – c) do neither show unspecific binding of ^{His}-eGFP or ^{His}-eGFP_DTA to cellular surface nor staining of unspecific structures after incubation with GFP-Booster. All respective mutants (d – f) show binding to primary rat lung fibroblasts. Moreover, zooms of distinct ^{His}-eGFP_CRM197 areas (white squares) reveal significant close proximity of murine HB-EGF (red) and ^{His}-eGFP_CRM197 mutants (green). A statistical evaluation (N = 3 cells) of detected green spots is displayed below each subimage.

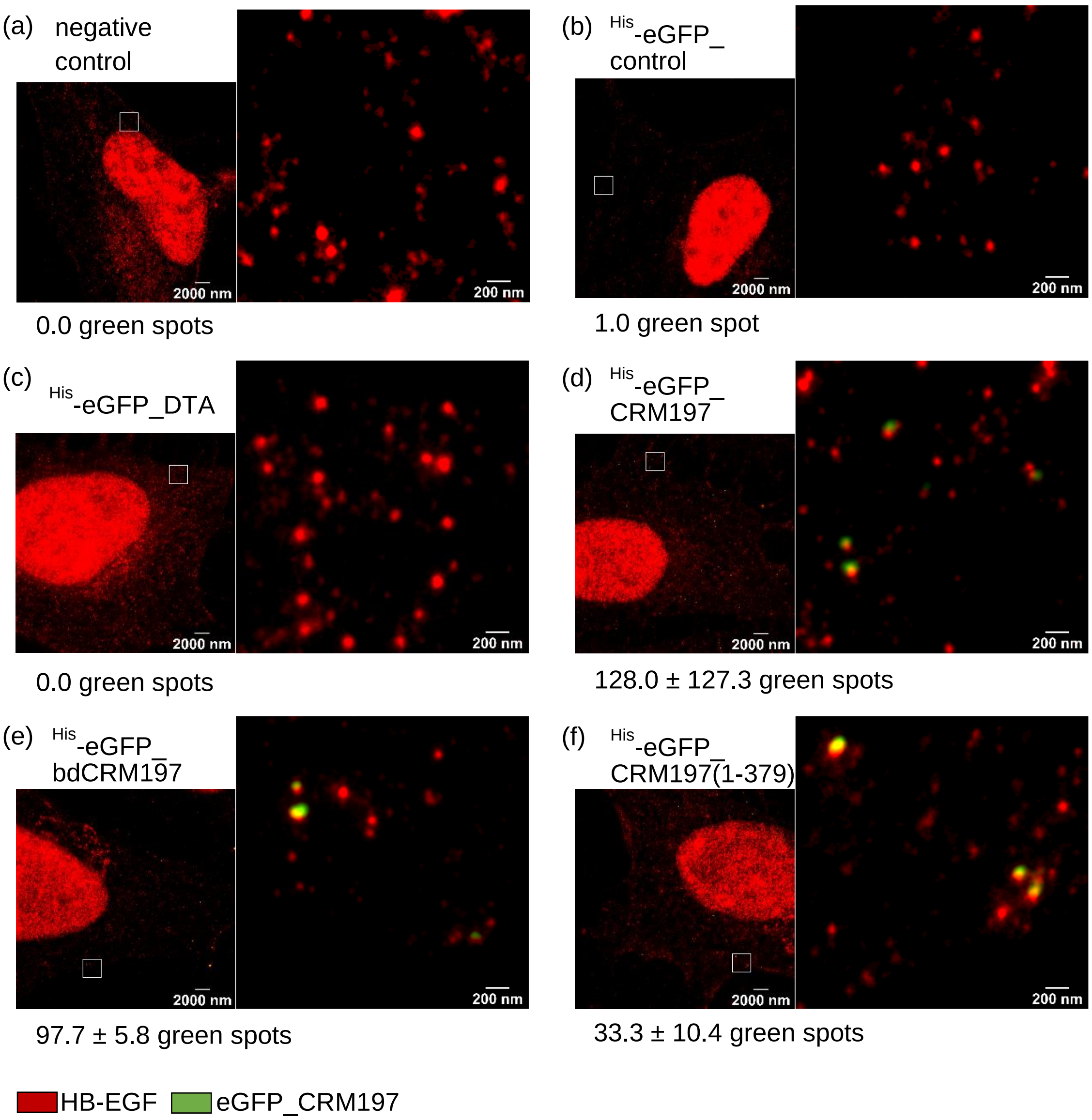

Fig. S 8

Human HeLa cells displaying bound ^{His}-eGFP_CRM197 mutants in close proximity to human HB-EGF captured by STED microscopy. (b) ^{His}-eGFP, (c) ^{His}-eGFP_DTA, (d) ^{His}-eGFP_CRM197, (e) ^{His}-eGFP_bdCRM197 as well as (f) ^{His}-eGFP_CRM197(1-379) (250 nM each) were incubated on human HeLa cells for 30 min at 4 °C. Negative control, ^{His}-eGFP control and ^{His}-eGFP_DTA (a – c) do neither show unspecific binding of ^{His}-eGFP or ^{His}-eGFP_DTA to cellular surface nor staining of unspecific structures after incubation with GFP-Booster. All respective mutants (d – f) show binding to human HeLa cells. Moreover, zooms of distinct ^{His}-eGFP_CRM197 areas (white squares) reveal significant close proximity of human HB-EGF (red) and ^{His}-eGFP_CRM197 mutants (green). A statistical evaluation (N = 3 cells) of detected green spots is displayed below each subimage.

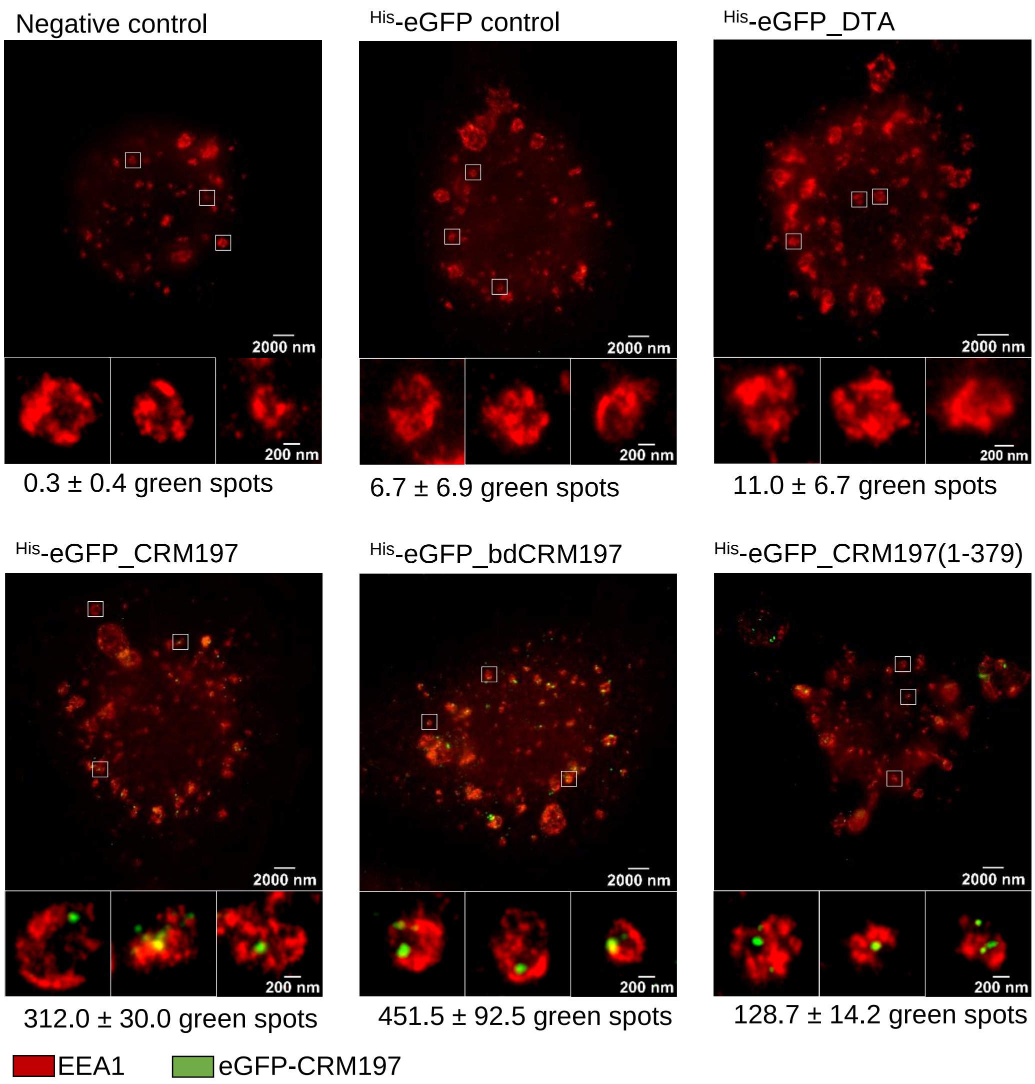

Fig. S 9

Mouse J774A.1 macrophages showing early endosomal antigen 1 (EEA1) (red) and His-eGFP_CRM197 mutants (green) captured with STED super-resolution microscopy. Incubation of ^{His}-eGFP, ^{His}-eGFP_DTA, ^{His}-eGFP_CRM197, ^{His}-eGFP_bdCRM197 as well as ^{His}-eGFP_CRM197(1-379) (250 nM each) for 30 min at 37 °C exhibit internalization of ^{His}-eGFP_CRM197 mutants (green) into early endosomes (red). Moreover, zooms of distinct ^{His}-eGFP_CRM197 signals (white squares) reveal detailed structural information. Equally, negative control, ^{His}-eGFP and ^{His}-eGFP_DTA do neither show immunostaining of unspecific structures nor internalization of ^{His}-eGFP or ^{His}-eGFP_DTA after incubation for 30 min at 37 °C. A statistical evaluation (N = 3 cells) of detected green spots is displayed below each subimage.

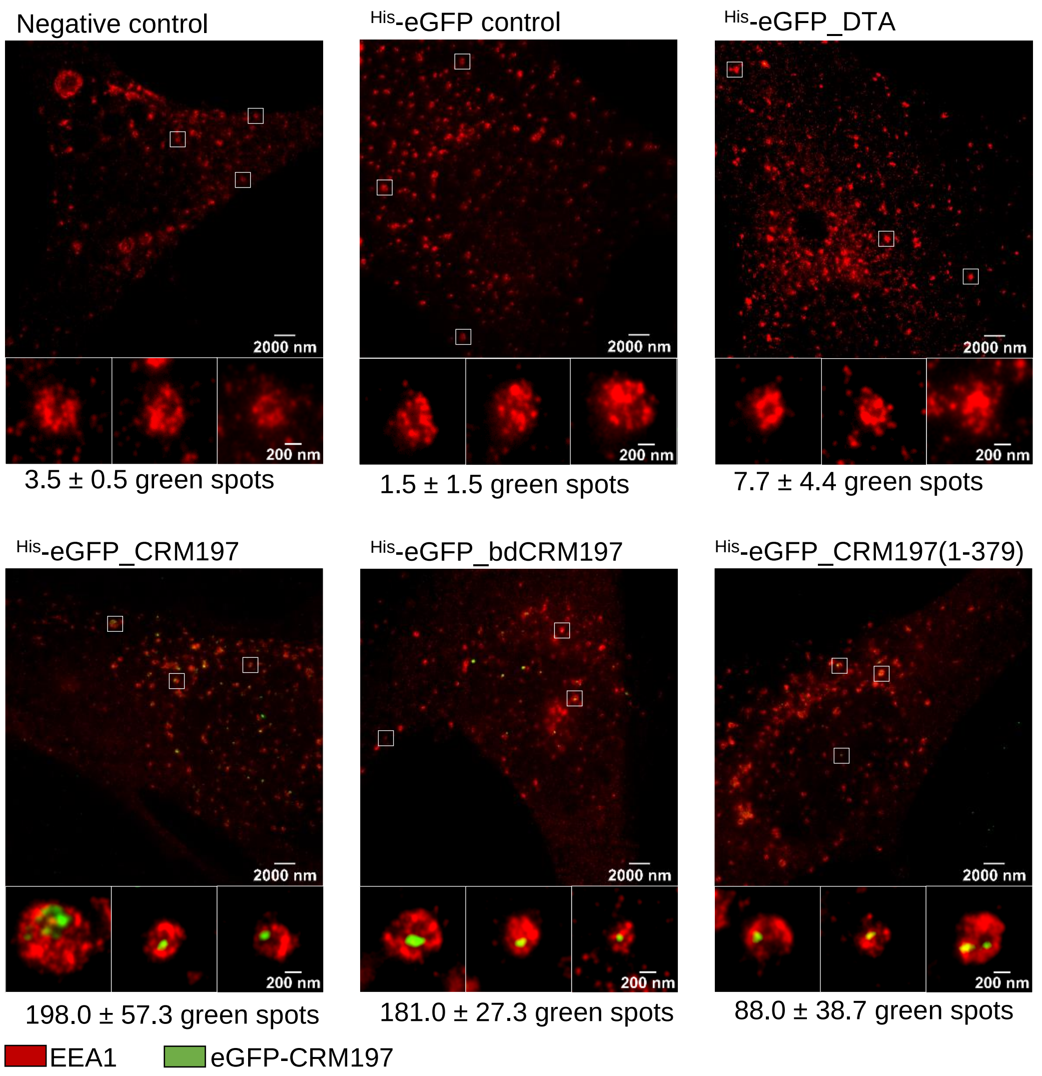

Fig. S 10

Primary rat lung fibroblasts showing early endosomal antigen 1 (EEA1) (red) and His-eGFP_CRM197 mutants (green) captured with STED super-resolution microscopy. Incubation of ^{His}-eGFP, ^{His}-eGFP_DTA, ^{His}-eGFP_CRM197, ^{His}-eGFP_bdCRM197 as well as ^{His}-eGFP_CRM197(1-379) (250 nM each) for 30 min at 37 °C exhibit internalization of ^{His}-eGFP_CRM197 mutants (green) into early endosomes (red). Moreover, zooms of distinct ^{His}-eGFP_CRM197 signals (white squares) reveal detailed structural information. Equally, negative control, ^{His}-eGFP and ^{His}-eGFP_DTA do neither show immunostaining of unspecific structures nor internalization of ^{His}-eGFP or ^{His}-eGFP_DTA after incubation for 30 min at 37 °C. A statistical evaluation (N = 3 cells) of detected green spots is displayed below each subimage.

Supplementary references

Blanke SR, Milne JC, Benson EL, Collier RJ (2002) Fused polycationic peptide mediates delivery of diphtheria toxin A chain to the cytosol in the presence of anthrax protective antigen. Proc Natl Acad Sci 93:8437–8442. https://doi.org/10.1073/pnas.93.16.8437

Goor RS, Pappenheimer AM, Ames E (1967) Studies on the mode of action of diphtheria toxin. V. Inhibition of peptide bond formation by toxin and NAD in cell-free systems and its reversal by nicotinamide. J Exp Med 126:923–39. https://doi.org/10.1084/jem.126.5.923

Moehring JM, Moehring TJ (1968) The response of cultured mammalian cells to diphtheria toxin. II. The resistant cell: enhancement of toxin action by poly-L-ornithine. J Exp Med 127:541–54
